# What Does the “AKT” Stand for in the Name “AKT Kinase”? Some Historical Comments

**DOI:** 10.3389/fonc.2020.01329

**Published:** 2020-08-11

**Authors:** Jiuyong Xie, Ralf Weiskirchen

**Affiliations:** ^1^Department of Physiology and Pathophysiology, Rady Faculty of Health Sciences, Max Rady College of Medicine, The University of Manitoba, Winnipeg, MB, Canada; ^2^Institute of Molecular Pathobiochemistry, Experimental Gene Therapy and Clinical Chemistry (IFMPEGKC), RWTH University Hospital Aachen, Aachen, Germany

**Keywords:** AKT, kinase, name, origin, oncogene, AKR mice, retrovirus

The Akt serine/threonine kinase family is comprised of three highly homologous isoforms, namely Akt1 (PKBα, OMIM: 164730), Akt2 (PKBβ, OMIM: 164731), and Akt3 (PKBγ, OMIM: 611223). They are stimulated by a large variety of extracellular stimuli. Individual AKT members have a widely diverse repertoire of downstream effects in different settings by targeting over 100 different substrates (1). The origin of “Akt” in the “Akt kinase” (also called protein kinase B or PKB) is explained here based on the original research papers describing the related information tracing back to 1933.

Prompted by the “a serine/threonine protein kinase” put for the abbreviation AKT kinase by a student, we searched for the proper meaning of “AKT” but no clear answer was found, to our surprise. Further search in the literature resulted in the following findings:

1. The 1987 paper by Dr. Stephen P. Staal of the Johns Hopkins Oncology Centre, on “*Molecular cloning of the akt oncogene and its human homologues AKT1 and AKT2: Amplification of AKTI in a primary human gastric adenocarcinoma*” (2), mentioned “the isolation of a directly transforming retrovirus, AKT8 from a spontaneous thymoma of an AKR mouse.” Going back 10 years, it is further mentioned that the initial isolate of the virus strain T-8 was from “an *in vitro* thymoma cell line, AKT-8, from a spontaneously lymphomatous AKR/J mouse” (3). The “thymoma” interpretation for the virus name AKT8 was also noted later as “for AKR Thymoma #8” by Bellacosa et al. in a 2005 review (4). Therefore, the “AK” was likely carried from the “AKR” of the mouse name, and the “T” was for the word “thymoma” describing the cellular source of the retrovirus, though it could also remind us of the “transforming” ability of the virus. The viral oncogene isolated from the AKT-8 was named v-*akt*. Therefore, the letters of the gene likely stand for the same.
2. The name AKR for the mouse strain was specifically explained by Dr. Clara J. Lynch of the Rockefeller Institute for Medical Research in a 1954 paper “*The R.I.L. strain of mice: its relation to the leukemic AK strain and AKR substrains*” (5). She explained: “The appellation AKR has now been adopted for the substrains to indicate the derivation of the random-bred colony from AK stock and the subsequent brother × sister breeding at the Rockefeller Institute,” in line with the recommendations by the Committee on Standardized Nomenclature for Inbred Strains of Mice (6). Therefore, the “R” in “AKR” should stand for Rockefeller Institute. Dr. Lynch also mentioned the “AK stock of Dr. J. Furth,” suggesting that the letters “AK” was from Dr. Furth. In addition, she mentioned in her manuscript “From a random-bred stock in the laboratory of Dr. Cornelius P. Rhoads at the Rockefeller Institute, brother × sister inbreeding of lines selected for leukemia was begun by Katherine B. Rhoads” and further “Data on the early generations, presented through the courtesy of Dr. C. P. Rhoads and K. B. Rhoads, are given in text-Figure 1” (5). So interestingly the “R” in “AKR” coincides with the letter R in “random” or “Rhoads.”
3. The meaning of “AK” was explained in a 1933 paper “*Experimental studies on lymphomatosis of mice*” by Dr. J. Furth et al. of the Cornell University Medical College and the University of Pennsylvania (7). It said: “….mice of three different stocks bred by us and designated as A, R, and S,” followed later by “…. the inbred families (of each stock) are designated by a second small letter added to the capitals A, R, and S respectively (e.g., Aa, Ab, etc., Ra, Rb, etc.).” One of the transmissible strains is named Ak30, as described later in the paper. The meanings of A, R, etc. were not found to be explained there. The authors simply explained “Mice colonies studied: The spontaneous cases of lymphomatosis that will be described are derived from mice of three different stocks bred by us and designated A, R, and S. Stock A was purchased because it was claimed to yield many cancers, stock R because it was stated to be non-cancerous. No information was obtained concerning stock S.” So the stock A was taken because it's high susceptibility for tumor formation.
4. The full length v-*ak*t oncogene was cloned, sequenced and biochemically characterized as a protein kinase C-related serine-threonine kinase by the Staal group in 1991 (8). Mammalian counterparts of this oncogene were isolated, sequenced and characterized independently by the Hemmings group in Switzerland and by the Woodgett group in England in the same year (9, 10). The former isolated them by cDNA library screening from porcine kidney LLC-PK_1_ cells using a porcine cAMP-PK probe, and then from human epithelial MCF-7 or lung fibroblast WI38 cell line (9, 11). The latter did so from human fibroblast using an amplified cDNA probe with degenerate oligonucleotide primers designed from regions conserved in the serine/threonine protein kinase catalytic domains (10). They were termed rac kinases (related to the A and C kinases) (9) or PKB (for Protein Kinase B most similar to the PKC/PKA families) (10).

Taken together these points, it seems that the origin of the name for the mouse homologue AKT of the viral v-*akt* gene product could be at least interpreted as “a serine/threonine protein kinase encoded by the oncogene in the transforming retrovirus isolated from the thymoma cell line AKT-8, which is derived from the Stock A Strain k AKR mouse originally inbred in the laboratory of Dr. C. P. Rhoads by K. B. Rhoads at the Rockefeller Institute.” Same interpretation applies to the human AKT kinases and genes.

## Author Contributions

JX and RW wrote the commentary together. All authors contributed to the article and approved the submitted version.

## Conflict of Interest

The authors declare that the research was conducted in the absence of any commercial or financial relationships that could be construed as a potential conflict of interest.
